# Cognitive Remediation Interventions in Schizoaffective Disorder: A Systematic Review

**DOI:** 10.3389/fpsyt.2018.00470

**Published:** 2018-10-04

**Authors:** Ester Lopez-Fernandez, Brisa Sole, Esther Jimenez, Estela Salagre, Anna Gimenez, Andrea Murru, Caterina del Mar Bonnín, Benedikt Lorenz Amann, Iria Grande, Eduard Vieta, Anabel Martínez-Aran

**Affiliations:** ^1^Unidad de Patología Dual, Hospital Santa Maria, Gestió de Serveis Sanitaris, Lleida, Spain; ^2^Barcelona Bipolar Disorders Program, Institute of Neurosciences, IDIBAPS, CIBERSAM, Hospital Clinic, University of Barcelona, Barcelona, Spain; ^3^Institute of Neurosciences, Hospital Clinic, University of Barcelona, Barcelona, Spain; ^4^Centro Fórum Research Unit, CIBERSAM, Institut de Neuropsiquiatria i Addiccions, Hospital del Mar, Barcelona, Spain; ^5^Department of Psychiatry, Institut Hospital del Mar d'Investigacions Mèdiques, Barcelona, Spain; ^6^Department of Psychiatry, Autonmous University Barcelona, Barcelona, Spain

**Keywords:** schizoaffective disorder, affective psychosis, cognitive enhancement, cognitive remediation, cognitive rehabilitation, cognitive training

## Abstract

**Background:** Patients with schizoaffective disorder (SAD) suffer from cognitive impairment, which negatively influences their functionality. Cognitive remediation (CR) interventions have been shown to be effective in patients with schizophrenia (SZ) and bipolar disorder (BD), but evidence in SAD is limited so far. The aim of this study is to systematically review the published data on CR interventions, either in neurocognition or social cognition, in patients with SAD.

**Methods:** We conducted a comprehensive, computerized literature search using terms related to CR interventions in psychotic and affective disorders, and particularly in SAD. Pubmed, Embase, and Web of Knowledge databases were used up to February 28th, 2018 according to the Preferred Reporting Items for Systematic Reviews and Meta-Analyses (PRISMA) statement. The search returned 2672 articles of which four were finally selected meeting the inclusion criteria.

**Results:** Cognitive Enhancement Therapy, computerized Cognitive Remediation Therapy and Cognitive Training showed positive results in subsamples of patients with SAD regarding neurocognition and functioning in comparable terms to patients with schizophrenia as well as in a greater extent in quality of life. Benefits in social cognition were also described when Social Cognition Interaction Training was considered in patients with SAD.

**Conclusions:** CR interventions seem to improve neurocognition and social cognition in patients with SAD as well as functioning and quality of life. However, further randomized controlled trials on CR interventions with an optimized design focusing on selected sample of patients with SAD are imperative.

## Introduction

Cognitive impairment is highly prevalent in several mental disorders, especially in those presenting with psychotic symptoms (1–5). Therefore, a neuropsychological examination of patients with psychiatric disorders has been progressively integrated in the elementary assessment of these patients (6, 7). Cognitive impairment has been widely studied in patients with schizophrenia (SZ), who usually exhibit some cognitive dysfunction preceding the illness onset (8). The most prevalent impaired cognitive domains in these patients are attention, processing speed, working memory, and problem solving (9–11). Cognitive impairment is also common in bipolar disorder (BD) even during euthymia (12–15). Although a subgroup of patients with BD may present some mild cognitive deficits before illness onset or even a higher cognitive performance than healthy population, most patients present an average cognitive performance until the first episode (16–18). After illness onset, cognitive performance in BD declines in particular in the domains of attention, verbal learning and memory, and executive functions according to clinical severity and number of relapses (8, 13, 19). Therefore, in general terms, there are many similarities between SZ and BD including scope of cognitive domains (20, 21).

First descriptions on cognitive performance of patients with schizoaffective disorder (SAD) come from studies with mixed samples of patient with SAD and SZ (22–24). Later, comparisons on the cognitive performance between SAD and SZ were also published (25–33). On the one hand, studies suggested that both groups of patients might present a similar pattern of neurocognitive impairment, especially in memory, executive functions, cognitive flexibility, reasoning, and problem solving (25–28). On the other hand, subsequent studies described less severity of neurocognitive impairment in patients with SAD compared to patients with SZ (29–33). Concerning social cognition, patients with SAD displayed a higher performance on tasks related to the Theory of Mind (ToM) compared to patients with SZ (32). When comparing the neurocognitive performance between patients with SAD and BD, poorer execution in verbal memory and occupational functioning has been detected in patients with SAD (4). All in all, these findings evidence the cognitive heterogeneity in patients with SAD (31, 34) and place this disorder in an intermediate position in terms of cognitive performance between SZ and BD although possibly closer to SZ (35). In terms of structural neuroimaging abnormalities, SAD also resembles more SZ than BD (36).

Since cognitive impairment is related to a worse clinical course and poor functional outcome (3, 37–40), it needs to be considered as a therapeutic clinical target in order to improve both psychosocial functioning and quality of life of patients with SAD (41–44). Nowadays some studies have suggested that social cognition may explain more functional outcome variance than neurocognition and that is why social cognition has been increasingly considered as another important treatment target (45, 46). Cognitive remediation (CR) interventions in psychiatric disorders are psychological or pharmacological based approaches (42). Concerning pharmacological treatments in affective and psychotic disorders, evidence so far suggests only a small effect on cognitive improvement; several drugs with potential pro-cognitive effects are currently being investigated (47, 48). With regards to psychological approaches, CR interventions have been developed to improve cognitive processes such as attention, memory, executive function, social cognition, and metacognition (Cognitive Remediation Experts Workshop, April, 2010) (49, 50).

The evidence of CR in neurocognition and social cognition in patients with SAD mainly stems from mixed sample studies, generally of patients with SAD and SZ or in fewer cases patients with SAD and BD (51). Although there are no studies focused exclusively on analyzing the efficacy of cognitive interventions in samples composed by patients with SAD, a systematic review about cognitive rehabilitation on patients with SAD as well as affective disorders hinted an improvement on the level of cognitive performance after completion of cognitive remediation in patients with SAD (52). The data of SAD in this study were determined by estimated pooled effect size (ES) weighted for the percentage of patients with SAD. Potential changes in other outcomes apart from cognition, such as social cognition, psychosocial functioning, and quality of life were not analyzed. According to the lack of knowledge of CR interventions in patients with SAD, we aimed to systematically review the evidence on CR interventions in neurocognition, social cognition, psychosocial functioning, and quality of life in patient with SAD exclusively and describe their possible benefits in these particular patients.

## Methods

This systematic review was conducted following the Preferred Reporting Items for Systematic reviews and Meta-Analyses (PRISMA) guidelines (53).

### Data sources and search terms

A comprehensive literature search of CR interventions in SAD was conducted by three authors independently (EL, BS, and IG) using the search terms in Pubmed, Embase, and Web of Science electronic databases from inception to February 28th, 2018.

The following Boolean logic algorithms were used: In Pubmed, (schizoaffective OR schizo-affective OR “affective disorder” OR “affective psychosis” OR “bipolar” OR “manic depression” OR schizophrenia OR “schizophreniform psychosis”) AND (“cognition training” OR “cognition therapy” OR “cognitive remediation” OR “cognitive training” OR “cognitive rehabilitation” OR “cognitive therapy” OR “cognitive intervention” OR “cognitive treatment” OR “neurocognitive remediation” OR “neurocognitive training” OR “neurocognitive rehabilitation” OR “neurocognitive therapy” OR “neurocognitive intervention” OR “neurocognitive treatment” OR “neuropsychological training” OR “neuropsychological rehabilitation” OR “neuropsychological therapy” OR “neuropsychological treatment” OR “metacognitive training”); and in Embase and Web of Science: “schizoaffective AND (“cognitive remediation” OR “cognitive rehabilitation” OR “cognitive training”).

Reference list of individual papers were also examined to identify any additional relevant studies.

### Study inclusion criteria

Records were reviewed using the following inclusion criteria: (1) Published studies (randomized clinical trials and follow-up cohort studies) about cognitive interventions targeted at improving cognitive skills, functioning, or quality of life which reported results about the sample or subsample of patients with SAD with at least 2 timing outcomes measures; (2) number or proportion of cases diagnosed with SAD in the sample; (3) diagnoses of SAD according to DSM-III, DSM-III-R, DSM-IV, DSM-IV-TR, DSM-5, ICD-9, or ICD-10; (4) no language restrictions were applied in this review; (5) no comparator group was imperative.

### Study exclusion criteria

The exclusion criteria applied were: (1) meta-analyses, systematic or narrative reviews, single cases, cases series, study protocols, letters to the editor, editorials, debate articles, opinion papers or congress abstracts; (2) interventions not involving CR interventions; (3) trials without identifying the number of participants with SAD; (4) studies without concrete outcomes about patients with SAD.

### Procedures and data extraction

Articles were selected based on title and abstract and, when necessary, on examination of the full text to assess its relevance. After elimination of duplicated sources, the full texts of the potentially eligible studies were considered. References were also reviewed to identify further possible studies of interest. Most existing articles on this subject about patients with psychosis and BD were reviewed, since in many cases the sample was mixed and the diagnosis of SAD was not detected in the search.

Extracted information was synthesized in two tables. In Table 1 the characteristics of the selected studies and main results are summarized: (a) first author and year of publication; (b) characteristics of the sample: (c) sample diagnosis; (d) study design; (e) outcome measures; (f) results summary; and (g) limitations. In Table 2 the characteristics of the interventions applied according to the following structure: (a) intervention; (b) target; (c) duration; (d) setting: individual or group intervention; and (e) type: computer assisted or non-computer assisted sessions.

**Table 1:** Characteristics of the studies selected on Cognitive Remediation interventions in schizoaffective disorder.

**Study**	**Sample characteristics**	**Sample diagnosis (n)**	**Design**	**Outcome measures**	**Results summary**	**Limitations**
Lahera et al. (54)	37 outpatients Age = 39.2 (10.4) years old Gender = 64.9% female Illness = 13.3 (7.8) years	BDI = 28 BDII = 5 SAD = 4	SCIT vs. TAU *n* = 21/16 SAD: 14.3% /6.3% Quasi-experimental study	**Social cognition** Emotion cognition: FEIT and FEDT Emotion recognition: ER40 ToM: Hinting task Social cognitive biases: AIHQ **Psychosocial functioning**: FAST and GAF	Significant group effects on every social cognitive outcome measure except for the AIHQ Intentionality subscale. No evidence of effects on aggressive attributional biases or on global functioning. Similar pattern of results with SAD excluded except no longer a significant group effect on AIHQ Intentionality or FEIT scores.	Quasi-experimental design (5 subjects reassigned after random) Heterogeneous sample No follow-up assessment
Lewandowski et al. (8)	58 outpatients Age = 25.9 (6.3) years old Gender = 31.0% female Illness = 3.2 (2.2) years	SZ = 38 SAD = 20 (12 depressed type, 8 bipolar type)	CET vs. EST *n* = 31/27 SAD: 32.3%/37.0% Subanalysis of RCT	**Processing speed:** Simple reaction time, choice reaction time and Visual-spatial scanning **Neurocognition:** WMS-R, California Verbal Learning Test, WAIS-R, TMT B, Wisconsin card sorting test, Tower of London, Neurological evaluation scale **Cognitive style:** Cognitive style and social cognition eligibility interview, Cognitive styles inventory **Social cognition:** Mayer-Salovey-Caruso Emotional Intelligence Test, social cognition profile, Cognitive style and social cognition eligibility interview **Social adjustment:** Social adjustment scale-II, Major role inventory, Global assessment scale, performance potential inventory, DHHS	SZ and SAD improved in multiple neurocognitive and social cognition domains after CET. Diagnosis did not significantly moderate this improvement. SAD had less improvement on neurocognition and cognitive style than SZ. No significant effects in processing speed. SAD groups exhibited significantly greater improvement on symptoms, specifically on depression and anxiety.	Small sample and unequal between groups Diagnostic stability was unclear in the sample (some patients changed symptoms over the study period)
Scheu et al. (56)	32 in- and outpatients Age = 33.4 (10.4) years old Gender = 46.9% female	SZ = 22 SAD = 10	cCRT (CogPack). 70 CogPack-tasks Retrospective study	**CRT response:** % of improved tasks based on the amount of completed tasks without initial ceiling effects. **Verbal intelligence:** MWT-B **Attention**: Test d2, parameter concentration performance (KL) **Verbal memory:** RBMT **Processing speed and executive functioning:** TMT	The improvement rate was 68% (improved tasks based on the amount of completed tasks without initial ceiling effects). No significant differences between SZ and SAD. No significant relationship between any of the baseline cognitive or symptom measures and improvement rates.	No control group Small sample Dichotomous primary outcomes Tasks assessment only three times
					Better baseline cognition was associated with a higher percentage of tasks with initial ceiling effects. Improvement from baseline to the second assessment after 4 weeks on all neurocognitive functions. Greater improvement in poor cognitive performance or higher values on the PANSS scores at baseline.	
Twamley et al. (57)	89 outpatients 51 study completers Age = 47.3 (9.8) years old Gender = 35% female Illness = 25.4 (19.2) years	SZ = 45 SAD = 39 PNOS = 5	CT+SP vs. SP Subanalysis of RCT	**Prospective memory**: MIST, **Attention and vigilance**: WAIS-III **Verbal Learning and memory**: HVLT-R **Executive functioning:** WCST-64 **Quality of life**: QOLI. **Functional capacity:** UPSA **Cognitive insight**: Beck Cognitive Insight Scale	CT associated improvement was correlated with worse baseline scores on measures of cognitive performance, symptom severity, functional capacity, and self-rated quality of life, cognitive problems, and strategy use. SAD got more improvement than SZ in subjective quality of life at 6 months.	Small sample Passive control group was treatment as usual No previous measures of motivation or cognitive improvement insight

*AIHQ, Ambiguous Intentions Hostility Questionnaire; BD, Bipolar Disorder; BDI, Bipolar Disorder Type I; BDII, Bipolar Disorder Type 2; CET, Cognitive Enhancement Therapy; cCRT, Computerized Cognitive Remediation Therapy; CT, Cognitive Training; d2, Aufmerksamkeits-Belastungs-Test; DHHS, Department of Health and Human Services; ER40, Emotion Recognition; EST, Enriched Supportive Therapy; FAST, Functioning Assessment Short Test; FEDT, Face Emotion Discrimination Test; FEIT, Face Emotion Identification Task; GAF, Global Assessment of Functioning scale; HVLT-R, Hopkins Verbal Learning Test-Revised; KL, Concentration Performance value; MIST, Memory for Intentions Screening Test; MWT-B, Mehrfachwahl-Wortschatz-Intelligenztest test B; PNOS, Psychosis Non Specified; QOLI, Quality of life; RBMT, Rivermead Behavioral Memory Test; RCT, Randomized Controlled Trial; SCIT, Social Cognition and Interaction Training; SD, Standard Desviation; SP, Standard Pharmacotherapy; SZ, Schizophrenia; SAD, Schizoaffective disorder; TAU, Treatment As Usual; TMT, Trail Making Test; TMT A, Trail Making Test A; TMT B, Trail Making Test B; ToM, Theory of Mind; UPSA, University of California Performance-Based Skills Assessment; WAIS-III, Wechsler Adult Intelligence Scale Third Edition; WAIS-R, Wechsler Adult Intelligence Scale-Revised; WCST-64, Wisconsin Card Sorting Test-64 Card Version; WMS-R, Wechsler Memory Scale-Revised*.

**Table 2:** Description of the studied Cognitive Remediation interventions in schizoaffective disorder.

**Intervention**	**Target**	**Duration**	**Setting**	**Type**
Cognitive Enhancement Therapy (CET)	Cognitive functions and social cognition	Biweekly sessions (60 h cognitive training + 45 h social cognition) for 24 months	Individual/group	Computer assisted and non-computer assisted sessions
computerized Cognitive Remediation Therapy cCRT (CogPack)	Cognitive function	50 min sessions twice a week over a maximum period of 8 weeks	Individual	Computer assisted
Cognitive Training (CT)	Cognitive function	2 h once a week for 12 weeks	Group	Non-computer assisted
Social Cognition and Interaction Training (SCIT)	Social cognition	1 h once a week for 18 weeks	Group	Non-computer assisted

## Results

**Figure 1:**
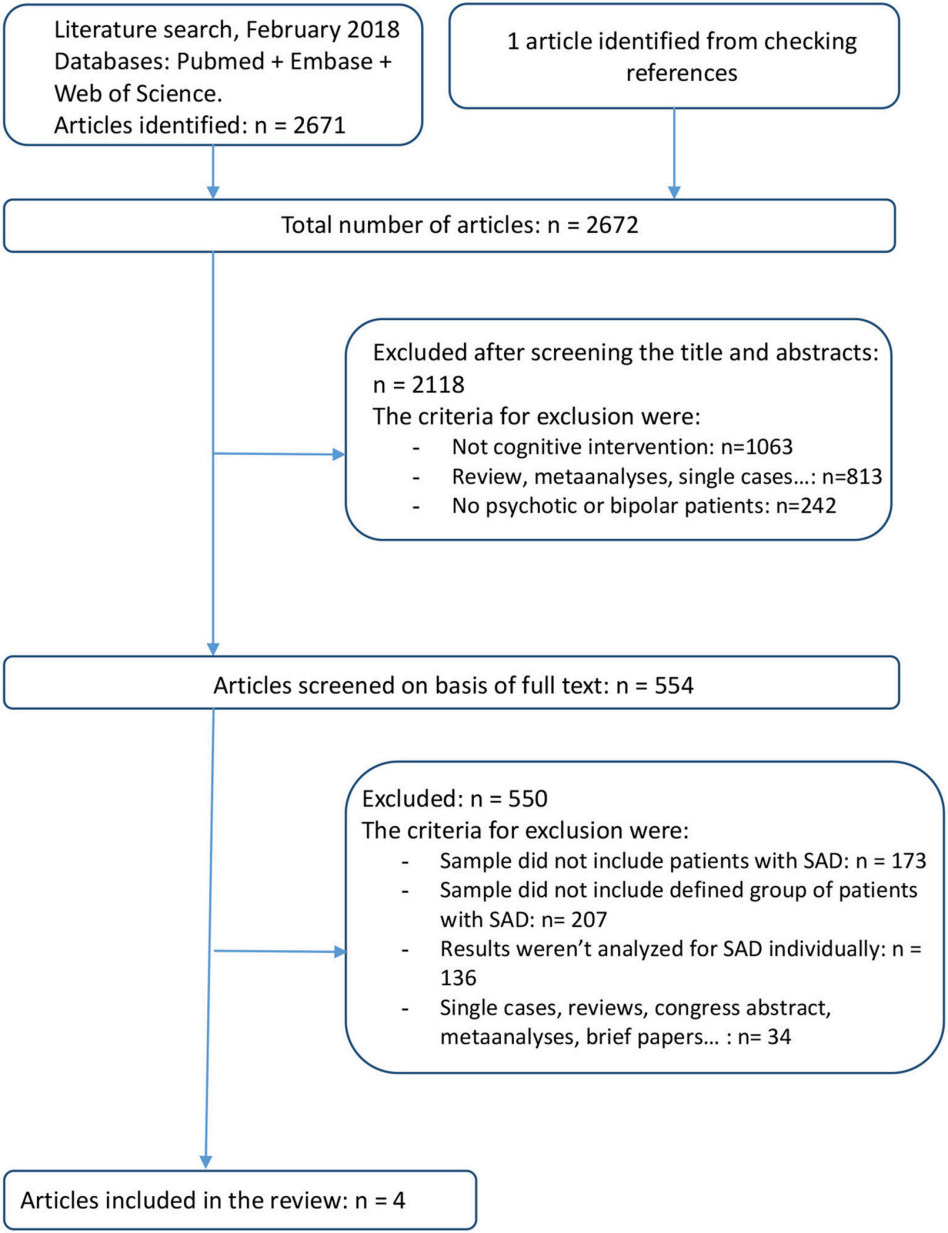
PRISMA flow-chart of the studies considered and finally selected for review.

Using the aforementioned keywords, the search returned 2672 records (Figure 1). The literature search identified 554 potentially relevant studies. After excluding studies that did not include or describe the sample of patients with SAD and their outcomes, four papers were identified according to the inclusion criteria (54–57).

The sample consisted of 73 patients with SAD out of 216 (Table 1).Two studies were performed in USA (55, 57), one in Germany (56), and one in Spain (54). The average study global sample size was 54 (SD 22.4) participants ranging from 32 to 89 patients. 58.3% of participants were men with a mean age of 38.1 (*SD* = 9.2) years. Three studies reported participants illness duration (54, 55, 57) which ranged from 3.2 to 30 years with a mean duration of 16.6 (SD 13.9) years. The average percentage of patients with SAD in the four studies was 33.8% in a range from 10.8 to 44%. The study with the largest sample of SAD was carried by Twamley et al. (57) with a sample of 39 patients. The interventions carried out in each study are described in Table 2.

Lewandowski et al. (55) compared a group that received Cognitive Enhancement Therapy (CET) with another group that received Enriched Supportive Therapy (EST) as a control group in a randomized controlled trial. The total sample included 20 patients with SAD and 38 with SZ. The authors conducted a secondary analysis comparing cognitive outcomes in patients with SAD and SZ with positive findings for CET in both diagnoses. The authors did not find a significant influence of the diagnosis on the relationship between improvement and treatment condition for the domains of processing speed, neurocognition, cognitive style, social cognition, social adjustment, or symptoms. Moreover, they described significant benefits for CET vs. EST for both SAD and SZ in within-group analysis: social cognition (SAD d = 1.69, SZ d = 1.68); social adjustment (SAD d = 1.36, SZ d = 1.65); and symptoms (SAD d = 1.00, SZ d = 0.68); all *p* < 0.045. In patients with SZ, CET produced significant improvement over EST in neurocognition (d = 0.46, *p* = 0.025) and cognitive style (d = 1.08, *p* = 0.009), however only trend-level effects were observed among patients with SAD (d = 0.52, *p* = 0.089 and d = 0.99, *p* = 0.098, respectively). No significant effect of the diagnosis on clinical improvement was found, with the exception of a significant reduction on depressive and anxious symptoms in patients with SAD (*p* = 0.019). This may be due to higher levels of anxiety and depression at baseline in this group of patients.

The computerized Cognitive Remediation Therapy (cCRT) is the intervention used in the study by Scheu et al. This study sample included 10 patients with SAD and 22 with SZ. After 4 weeks, the authors observed a significant improvement in the neurocognitive performance that involved attention memory, strategy, numeracy and visuo-motor skills in patients with SAD and SZ (56). No significant differences were found in improvement rates between both diagnostic groups. There was no significant correlation between improvement rates and the number of attended training sessions, but better improvement rates were linked to a higher total number of completed tasks (*r* = 0.36, *p* < 0.05). Correlation analyses revealed no significant relationship between any of the baseline cognitive or symptom measures and improvement rates. Cognitive improvements on processing speed and verbal memory were associated with higher baseline scores on the general PANSS and total PANSS (*r* = −0.44, *p* < 0.05; *r* = −0.45, *p* < 0.01, respectively), while improvements on Trail Making Test A were related to higher scores in the positive PANSS (*r* = −0.43, *p* < 0.05). Higher scores in the PANSS scores indicated worse clinical state.

Twamley et al. (57) studied the efficacy of Cognitive Training (CT) and Standard Pharmacotherapy (SP) compared to SP alone in a mixed sample of 39 patients with SAD, 45 with SZ and 5 with psychosis not otherwise specified. Patients showed a significant improvement in attention (*p* = 0.049), verbal memory (*p* = 0.017), and negative symptoms severity (*p* = 0.002) at 3-month follow-up and in verbal memory (*p* = 0.039), prospective memory (*p* = 0.050), functional capacity (*p* = 0.004), negative symptoms severity (*p* = 0.025), and self-reported quality of life (*p* = 0.004) at 6-month follow-up. Results of cognitive outcomes were not available according to diagnoses. However, patients with SAD showed a significant improvement in subjective perception of quality of life at 6 months compared to patients with SZ (*p* = 0.03) (57). At 3-month follow-up, improvement in digit span forward and in Hopkins Verbal Learning Test (HVLT) were associated with higher levels of negative symptoms severity at baseline (*r* = 0.45, *p* = 0.045; *r* = 0.50, *p* = 0.025, respectively). Moreover, improvement in digit span was related to higher levels of self-reported cognitive problems (*r* = 0.48, *p* = 0.033). An improvement in HVLT percent retention at 3 months was also associated with lower cognitive strategy use at baseline (*r* = −0.48, *p* = 0.033). At 6-month follow-up, improvement on the University of California, San Diego, Performance-Based Skills Assessment (UPSA) functional capacity was associated with higher levels of positive symptoms (*r* = 0.45, *p* = 0.035), lower levels of cognitive strategy use (*r* = −0.54, *p* = 0.009), and worse UPSA performance at baseline (*r* = −0.56, *p* = 0.007).

Lahera et al. (54) described the benefits of Social Cognition and Interaction Training (SCIT) compared to Treatment As Usual (TAU) in a mixed sample of 4 patients with SAD and 33 with BD. The authors detected a significant improvement in the group that received SCIT on each social cognitive outcome except for the Ambiguous Intentions Hostility Questionnaire (AIHQ) Intentionality subscale, with a trend to significance (*p* = 0.069). The group that received SCIT showed a significant improvement in emotion perception and ToM (*p* < 0.05), and significant improvement in hostile attribution biases compared to the TAU group (*p* < 0.05). The SCIT group showed a within-group improvement on the AIHQ Blame subscale (d = −0.19, *p* < 0.01), an improvement in AIHQ Hostility Bias (d = −0.55, *p* < 0.05), an improvement in scores on the Hinting Task (d = 0.4, *p* < 0.05), an improvement on the Emotion Recognition-40 (ER40) (d = 0.51, *p* < 0.05), and an improvement on the Face Emotion Discrimination Task (FEDT) (d = 0.67, *p* < 0.01) and Face Emotion Identification Task (FEIT) (d = 0.81, *p* < 0.05). *Post-hoc* analysis did not evidence an effect of diagnoses on the results. No evidence for between-group effects on any clinical outcome was found.

The risk of bias was assessed in all eligible studies as recommended by the Cochrane Collaboration (58). However it was difficult to determine due to the heterogeneity of the study design and because the focus of this systematic review was beyond the main objectives of the selected articles.

## Discussion

Despite the scarce number of studies on the topic, there is evidence, although limited, of the effectiveness of CR interventions in patients with SAD. CET, cCRT, and CT showed positive results in cognition in the subsample of patients with SAD considering neurocognitive or functional parameters as well as outcomes related to quality of life. Benefits in social cognition were also described when SCIT as well as CET were considered in patients with SAD.

These results are in line with previous bibliography on the issue. Regarding neurocognition, Anaya et al. (52) described in their meta-analysis that CR interventions showed positive effects on cognition at post-intervention in patients with SAD as well as in patients with affective disorders with an ES of 0.32. Interestingly, the authors pointed out that the effect of CR interventions increased when the meta-analysis was limited to studies that included exclusively patients with SAD, obtaining a pooled ES weighted for the percentage of patients with SAD of 0.41. In addition, we also have found some evidence that schizoaffective patients could improve in specific measures of social cognition, social adjustment, symptoms and quality of life after receiving a CR intervention.

It is worth commenting on the studies that presented a relevant percentage of patients with SAD in the sample but did not specifically mention results of the subsample of patients with SAD. Considering neurocognition, In a subsequent article (59) of the one included in this systematic revision, Twamley et al. described general improvement in cognitive domains considering the entire sample. In another study with 53% of the sample diagnosed with SAD (60), computer-assisted cognitive rehabilitation showed greater improvement in neurocognitive performance, specifically in verbal memory and attention, and negative symptoms compared to a wait-list control group. Regarding social cognition, a recent systematic review that included studies with samples of patients with SAD and SZ (61) stated that interventions in social cognition could improve several domains related to affect recognition, ToM and social perception. However, the effect on attributional style and the relationship between improvement in social cognition and functioning were unclear. All in all, CR interventions in neurocognition and social cognition seem to be effective in the psychotic spectrum.

Whether patients diagnosed with SAD benefit from CR interventions more than SZ or less than BD is still open to question. Lewandowski et al. published the results of CET between patients with SAD and SZ in a subanalysis of a previous study (55, 62). Although positive results were described in both groups, a lower benefit of the treatment was observed in the cognitive performance of patients with SAD compared to those with SZ. This may be due to a ceiling effect since patients diagnosed with SZ present more cognitive impairment compared to patients diagnosed with SAD. The evidence suggests that the wider the cognitive impairment at baseline, the greater benefits can be obtained with CR interventions. It may be due to the fact that there is more room for improvement or because of an increased motivation (57). Nevertheless, in the study performed by Scheu et al. (56), outcomes of patients with SAD did not differ from those observed in patient with SZ, being positive in both disorders. Thus, despite the cognitive heterogeneity (31, 34), SAD may be placed in an intermediate position in terms of neurocognitive performance between SZ and BD although possibly closer to SZ (35).

There is controversy about how basal clinical state may impact on the results of CR and how CR may influence the clinical state. With regard to the former, on one hand, Wykes et al. (63) reported in a meta-analysis focused on CR in patients with SZ that the benefits were more significant in less symptomatic patients. On the other hand, Twamley et al. (57) found an association between higher levels of negative symptoms and greater benefits, and between higher levels of positive symptoms and greater improvement in functional capacity. Therefore, they consider that the presence or severity of symptoms should not be an exclusion criterion for these interventions. Other authors consider that the severity of positive or negative symptoms does not predict the rate of improvement (19, 56, 64). Considering the latter, the two meta-analyses by Wykes et al. and McGurk et al. (63, 65) described a significant positive effect of CR on both symptoms and functionality in patients with SZ. Lewandowski et al. (55) detected greater improvement in symptoms after receiving CET in patients diagnosed with SAD compared to patients diagnosed with SZ, specifically in anxious and depressive symptoms.

Another issue of debate is the right moment to provide CR interventions. Some authors suggest that the younger the patients, the more they benefit from CR interventions (63, 66–68, 70). On the contrary, the two major meta-analysis in the literature about CR interventions concluded no relationship between these two variables or that the older the patients, the better outcomes of CR interventions (63, 65). Twamley et al. pointed out that older patients achieved more improvement, specifically in prospective memory (57). The concept of cognitive reserve may provide an explanation for the discrepancy in these results since it reflects the capacity of the brain to endure neuropathology and successfully complete cognitive tasks (69). Moreover, cognitive reserve has been found as a significant predictor of cognitive and psychosocial functioning in patients with SZ and BD (70–72). Another key issue in CR interventions relates to the relationship between number of sessions and the obtained benefits. The meta-analysis carried out by Wykes et al. (63) and the study of Scheu et al. (56) did not reveal any association between the aforementioned variables. Last but not least, the drop-out ratio is another matter of concern in CR interventions. Twamley et al. (57) analyzed who was more likely to drop out in their randomized controlled trial of CT in which 57.30% of the patients completed the therapy while 31.46% did not start it and 11.24% withdrew. Those who completed CT had more formal education and lower antipsychotic doses than had dropouts with no CT exposure, but the groups did no otherwise differ. In Lewandowski et al. (55) and Lahera et al. (54) studies, the frequencies of dropouts were 20.6 and 19.1%, respectively.

As a summary, Lewandowski et al. (55) obtained small effects on neurocognition in the group of SAD, vs. medium effects in the group of SZ. However, patients with SAD improved more in symptomatology after cognitive treatment. In this study, a similar improvement in the functionality of both groups was obtained. On the other hand, Scheu et al. (56) did not find differences in improvement rates when comparing patients with SAD and SZ. Lahera et al. (54) did not find differences after treatment when compared patients with SAD and BD, considering that the sample included four patients with SAD. Twamley et al. (57) did not report group differences but more improvement in subjective quality of life at 6 months in SAD compared to patient with SZ.

Despite data gathered in this systematic review seems to support a positive effects of CR interventions in SAD, these results should be interpreted with caution. First of all, the samples of the four reviewed studies are restricted to small subsamples of patients diagnosed with SAD within a wider sample of patients diagnosed mostly with SZ or BD. Although we only consider articles that studied the concrete subsample of SAD, the obtained results stem from *post-hoc* analyses, which are not always aligned with the aim of the primary objective of the study and therefore may increase false positive results. Moreover, the heterogeneity of the design of the reviewed CR interventions should be beared in mind. This heterogeneity could partly explain discrepancies among results from these studies.

In this systematic review, scarce studies on CR interventions in SAD were found. However, available data support that CR interventions may improve neurocognition and social cognition in this group of patients. Subsequently, functioning and quality of life on this population may also benefit from improving the daily life of patients with SAD. So as to confirm this hypothesis, further randomized controlled trials on CR interventions with an optimized design and selected sample of patients with SAD are urged.

## Author contributions

All authors listed have made a substantial, direct and intellectual contribution to the work, and approved it for publication.

### Conflict of interest statement

IG has served as a consultant for Ferrer, advisor for Lundbeck, Otsuka and has been a speaker for Ferrer, Janssen and Lundbeck, Otsuka. EV has received grants and served as a consultant, advisor, or CME speaker for the following entities: AB-Biotics, Allergan, AstraZeneca, Bristol-Myers-Squibb, Ferrer, Forest Research Institute, Gedeon Richter, Glaxo-Smith-Kline, Janssen, Lundbeck, Otsuka, Pfizer, Roche, Sanofi-Aventis, Servier, Shire, Sunovion, Takeda, Telefonica, the Brain and Behaviour Foundation, the Spanish Ministry of Science and Innovation (Centro de Investigación Biomédica en Red de Salud Mental), the Seventh European Framework Programme (European Network of Bipolar Research Expert Centres), and the Stanley Medical Research Institute. The remaining authors declare that the research was conducted in the absence of any commercial or financial relationships that could be construed as a potential conflict of interest. The reviewer RS and handling editor declared their shared affiliation at time of review.
